# Global hotspots and trends in pre-metastatic niche research: a bibliometric analysis(2005-2024)

**DOI:** 10.3389/fimmu.2025.1552053

**Published:** 2025-05-29

**Authors:** Mengqi Cheng, Qianhui Sun, Hua Duan, Cihui Chen

**Affiliations:** ^1^ Department of Oncology, The First Affiliated Hospital of Zhejiang Chinese Medical University (Zhejiang Provincial Hospital of Chinese Medicine), Hangzhou, China; ^2^ Cancer Institute of Integrated Traditional Chinese & Western Medicine, Zhejiang Chinese Medical University, Hangzhou, China

**Keywords:** pre-metastatic niche, PMN, metastasis, bibliometric analysis, research hotspots, immunotherapy, metastatic organotropism, extracellular vesicles

## Abstract

**Background:**

The pre-metastatic niche (PMN) represents the microenvironment established in target organs before primary tumor metastasis, playing a crucial role in organ-specific metastasis. Understanding and preventing PMN formation holds promise for enhancing immunotherapy efficacy and reducing cancer-related mortality. Despite the significance of this field, a comprehensive bibliometric analysis is lacking. This study aims to identify global research trends and hotspots in PMN through a systematic bibliometric evaluation, providing a foundation for future advancements in this field.

**Methods:**

Publications related to PMN research from 2005 to 2024 were retrieved from the Web of Science Core Collection database. Bibliometric analyses and visualizations were conducted using VOSviewer, CiteSpace, Microsoft Excel, ArcGIS, Scimago Graphica, and Microsoft Charticulator.

**Results:**

The study included 1,303 publications authored by 7,955 researchers from 1,627 institutions across 62 countries, with articles published in 400 journals. China and the United States emerged as central contributors to global PMN research. China has led in publication volume and institutional representation, while the United States has produced the most high-quality papers and impactful authors. *Cancers* published the most PMN-related papers, while *Cancer Research* had the most citations and co-citations. Professor David Lyden of Cornell University, USA, was identified as the most influential scholar in the field. Analysis of references and keywords suggests future research will focus on metastatic organotropism, extracellular vesicles, innate immunocytes (e.g., macrophages and neutrophils), and immunotherapy.

**Conclusion:**

This bibliometric study represents the first comprehensive analysis of global scientific output in PMN research over the past two decades. By summarizing the current status and identifying trends in the field, this study provides valuable insights and a reference point for researchers aiming to prevent and treat tumor metastasis effectively.

## 1 Introduction

Tumor metastasis poses a significant challenge in oncology, serving as a primary cause of treatment failure and mortality among cancer patients (1). Biologically, metastasis involves tumor cells invading distant organs from their primary site via direct invasion, bloodstream dissemination, lymphatic spread, or adjacent structures (2). This process is driven by both the genetic characteristics of tumor cells, which enhance their metastatic potential, and specific alterations in the microenvironment of distant organs (3). Before circulating tumor cells (CTCs) reach target organs, factors produced by the primary tumor induce the formation of a “pre-metastatic niche” (PMN), a concept first proposed by David Lyden’s team in 2005 (4).

The PMN concept stems from Paget’s 1889 “seed-and-soil” theory, which suggests that tumor cells (seeds) metastasize in environments (soil) that promote colonization (5). Unlike the metastatic niche, which forms upon the arrival of CTCs, PMN represents a pro-tumorigenic microenvironment devoid of cancer cells (6). Emerging evidence highlights PMN’s pivotal role in organ-specific metastasis (7). For instance, primary pancreatic tumors are associated with heightened liver PMN inflammation and metabolic alterations that may indicate future liver metastasis (8). The formation of PMN requires tumor-derived secreted factors (TDSFs) and extracellular vesicles (EVs) to mediate changes in resident cells (e.g., macrophages, fibroblasts, endothelial cells). It also affects infiltrating immune cells (e.g., innate and adaptive immunocytes) within distant organs (9).

Together, these components create an inflammatory and immunosuppressive microenvironment. This microenvironment promotes the formation of new blood and lymphatic vessels (angiogenesis and lymphangiogenesis) and facilitates extracellular matrix (ECM) remodeling, ultimately enabling PMN formation (10).

As research advances, specific biomarkers of PMN and its organ-specific tendencies are becoming increasingly understood. Recent research increasingly focuses on clinical strategies to prevent and treat tumor metastasis, including inhibiting PMN-promoting signals, disrupting tumor-derived EV signaling, and modulating immune cell infiltration (11). Although previous reviews have addressed PMN research from various perspectives—such as formation, characteristics, and diagnostic or therapeutic potential (6, 9–14)—no comprehensive analysis highlights the evolution of PMN research or its future priorities.

Bibliometric analysis is a method that applies mathematical and statistical techniques to review systematically, and qualitatively and quantitatively analyze research within a specific field over a defined period (15). This study provides an objective overview of global PMN research through bibliometric analysis, integrating statistical data and visualization to highlight key contributors, institutions, and countries. We also explore research trends and future directions in the PMN field. Our findings aim to offer valuable insights for preventing PMN formation and metastasis while guiding researchers toward new research topics and strategic planning.

## 2 Materials and methods

### 2.1 Data retrieval and literature screening

**Figure 1:**
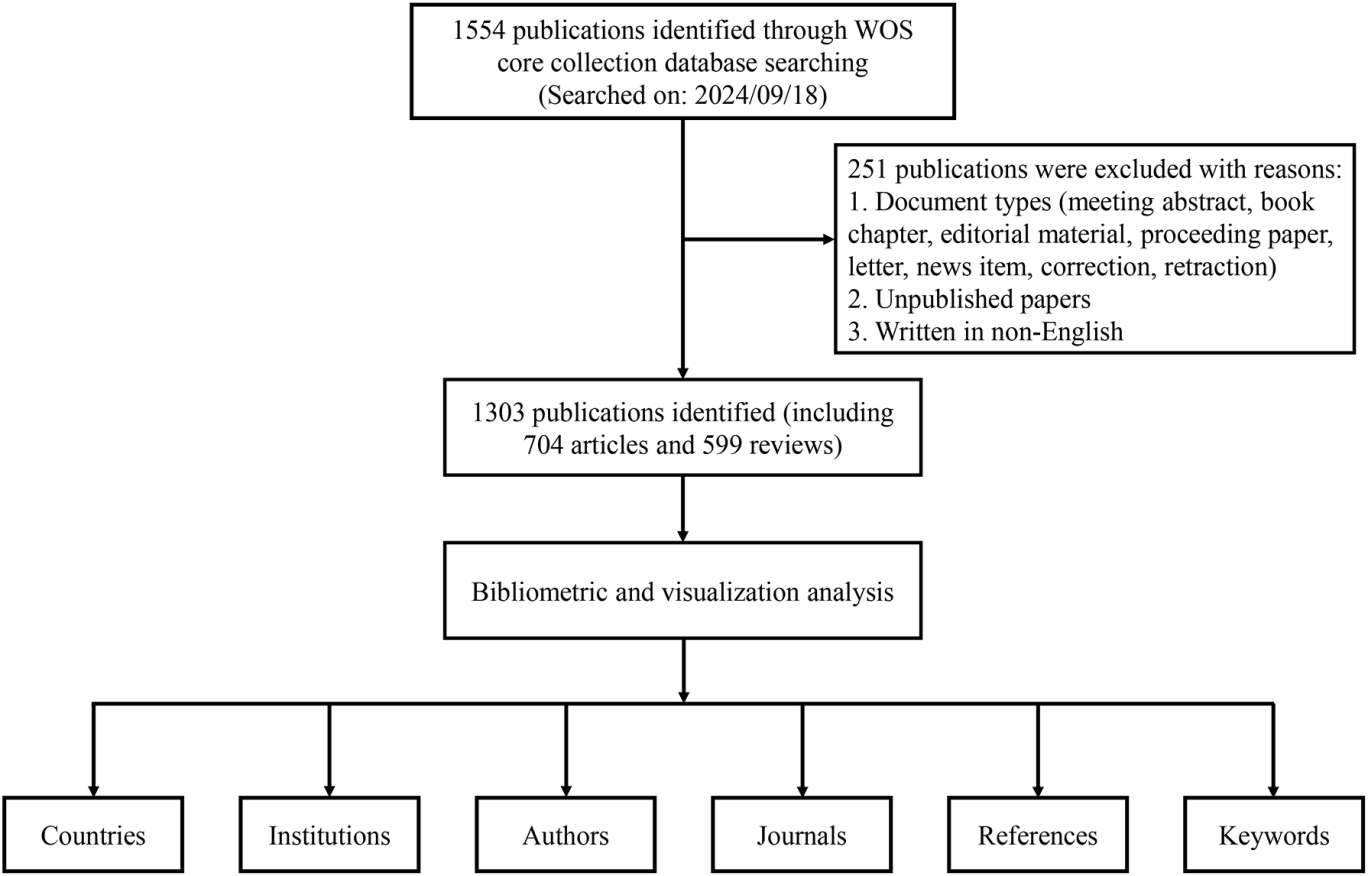
Detailed flowchart of data retrieval and literature screening.

All publication data for this study were obtained from the Web of Science Core Collection (WOSCC) database. In the WOSCC database, TS represents a topic search. The following search strategy was applied in this study: TS = (pre-metasta* OR premetasta*) AND TS = (niche* OR microenvironment*). The search period spanned January 1, 2005, to September 18, 2024. Original research articles and reviews published in English were included, while early-access papers and other document types were excluded (**Figure 1**). All data were downloaded as “plain text” files.

### 2.2 Data analysis and visualization

Two authors independently extracted bibliometric parameters, including titles, keywords, countries or regions, institutions, authors, journals, year of publication, citations, and cited references. The Impact Factor (IF) and H-index (16) were used to assess the academic influence of publications and researchers. Journal Citation Reports (JCR) 2023 was used to calculate the IF. A researcher’s H-index indicates that at least h papers have been cited at least h times. Additionally, the H-index was also used to evaluate the productivity and influence of countries, institutions, and journals.

VOSviewer 1.6.20 was employed to analyze and visualize the collected data, focusing on country cooperation, institutional and author cooperation, journals publishing and being cited, and keyword co-occurrence. Journal overlay dual maps, keyword time zone maps, and reference and keyword burst analyses were generated using CiteSpace 6.3.R3. In these visualizations, each node represents a distinct parameter, such as a country, institution, author, or keyword. The size of each node reflects its weight, which is determined by parameters like the number of publications (NP), number of citations (NC), or frequency of occurrence. A higher weight corresponds to a larger node. Nodes and lines were colored according to their cluster, while lines between nodes indicated links. The total strength of co-authorship and co-citation links between countries, institutions and authors was represented by the Total Link Strength (TLS).

Several additional tools were used to present and map the data. Microsoft Excel 2019 was used to depict publication and citation trends in the PMN field over the past 20 years. ArcGIS 10.8 facilitated the mapping of the geographic distribution of global publication output. Scimago Graphica 1.0.43 displayed the annual citation frequency of the top 10 articles based on total citations. Finally, Microsoft Charticulator (https://donghaoren.org/charticulator/index.html) was employed to create a chord diagram illustrating country cooperation.

### 2.3 Research ethics

All raw data used in this study were obtained from publicly accessible databases and did not involve animal or human experimentation. As such, no ethical approval was required.

## 3 Results

### 3.1 Publication output and citation trends

**Figure 2:**
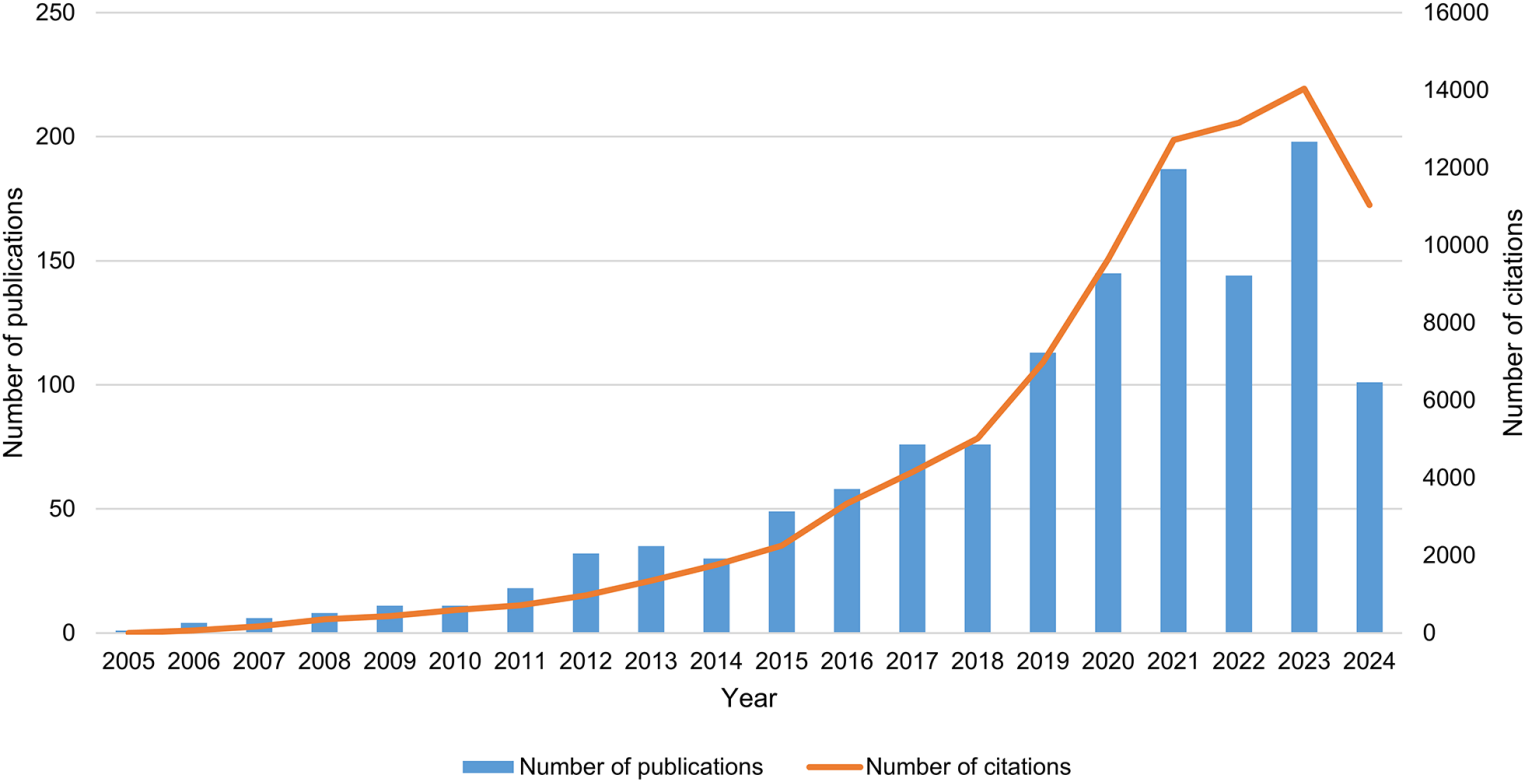
Trends in publication output and citations related to pre-metastatic niche.

As of September 18, 2024, a total of 1,303 articles on PMN were identified in the WOSCC database, comprising 704 original research articles and 599 reviews. Between 2005 and 2021, the NP in this field increased significantly (**Figure 2**). From 2022 to 2023, publication numbers fluctuated but trended upward, peaking in 2023 with 198 papers published (2024 data includes publications only up to September 18). Additionally, the annual NC has risen markedly over the past decade, reaching approximately 8,237.7 citations compared to an average of 641.1 citations during the first ten years of the study period. These trends indicate a growing interest in PMN research, highlighting the increasing academic value and potential impact of this field.

### 3.2 Distribution and cooperation of countries

**Figure 3:**
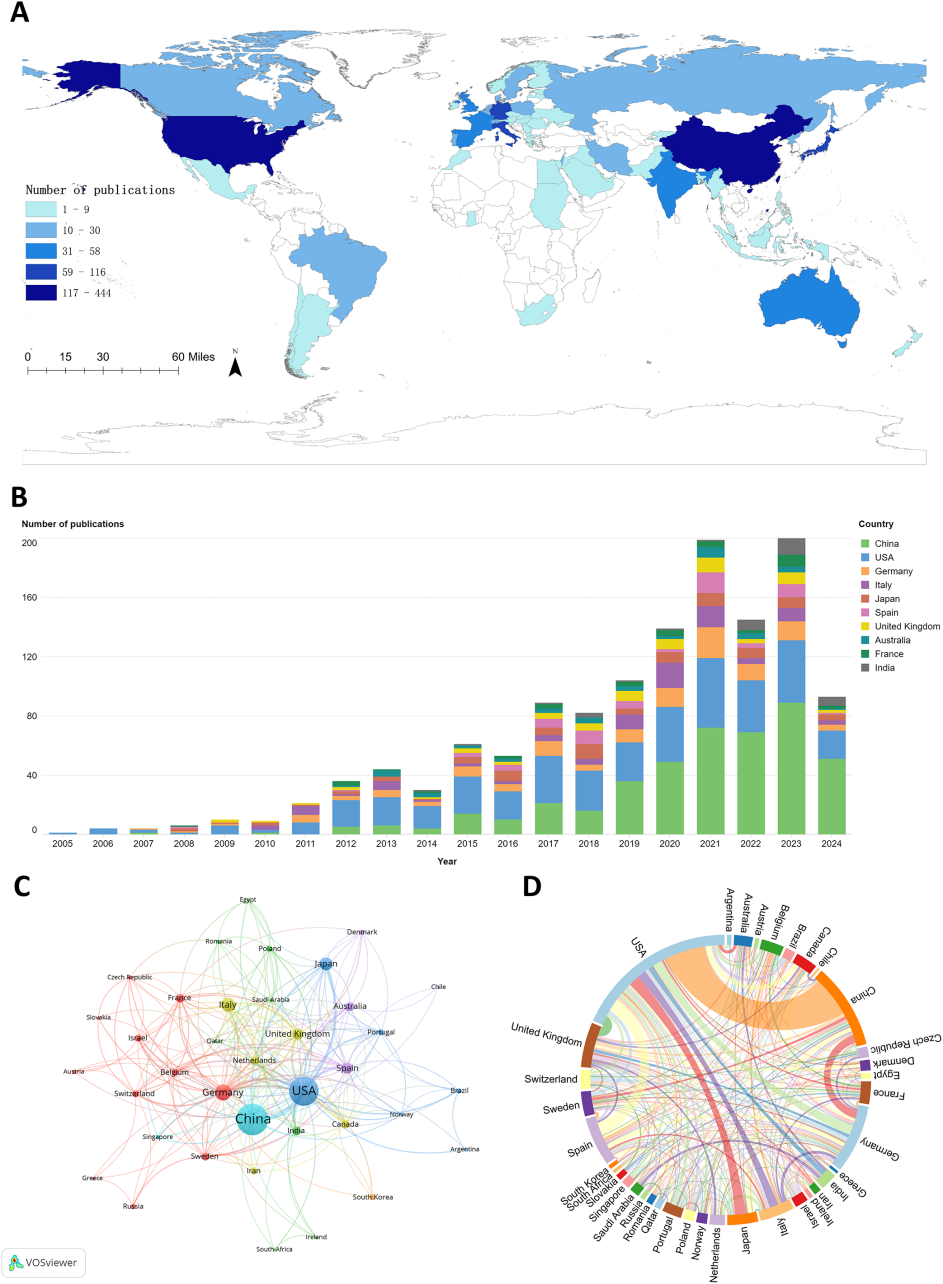
**(A)** The geographic distribution of global publications on pre-metastatic niches is shown in, highlighting publication density by country. **(B)** illustrates annual publication trends for the top 10 contributing countries. **(C)** presents the country cooperation network visualization map, generated using VOSviewer (version 1.6.20), depicting collaborations among countries through node-link relationships. **(D)** features the country cooperation chord diagram created with Microsoft Charticulator, where node size indicates the total number of collaborations for each country, and line thickness represents the frequency of collaboration between countries.

A total of 62 countries contributed to research in the PMN field. The geographic distribution of publications is presented in **Figure 3A**, where darker colors on the world map indicate higher numbers of publications. **Table 1** and **Figure 3B** rank the top 10 countries by the number of articles published on PMN. China accounted for the largest number of publications, contributing 34.1% (444/1,303) of the total, followed by the United States (29.5%, 385/1,303) and Germany (8.9%, 116/1,303). The United States had the highest total citations (48,655) and H-index (95), while articles from Spain achieved the highest average citations per article (229.91).

**Table 1:** The top 10 productive countries ranked by numbers of publications.

Rank	Countries	NP	N (%)	NC	AC	TLS	H-index
1	China	444	34.10%	23,856	53.73	128	68
2	USA	385	29.50%	48,655	126.38	243	95
3	Germany	116	8.90%	13,409	115.59	102	43
4	Italy	86	6.60%	4,232	49.21	52	32
5	Japan	76	5.80%	7,883	103.72	46	32
6	Spain	58	4.50%	13,335	229.91	73	29
7	United Kingdom	58	4.50%	9,550	164.66	67	31
8	Australia	40	3.10%	5,185	129.63	28	23
9	France	34	2.60%	1,660	48.82	34	19
10	India	33	2.50%	930	28.18	26	13

NP, number of publications; NC, number of citations; AC, average citations; TLS, total link strength; USA, The United States of America.

**Figure 3C** illustrates international collaboration among the top 37 countries, each with at least five publications. The collaboration network shows that the United States (TLS = 243), China (TLS = 128), and Germany (TLS = 102) collaborated most frequently with other countries, with the highest number of collaborations occurring between China and the United States (**Figure 3D**).

### 3.3 Distribution and cooperation of institutions

A total of 1,627 academic institutions contributed to PMN research. **Table 2** lists the top 10 institutions ranked by the number of publications, with institutions from the United States, China, and Germany dominating the list. Cornell University had the highest number of publications, closely followed by Sichuan University and Ruprecht Karls University Heidelberg. Cornell University also achieved the highest H-index, indicating its significant impact on the field, while Memorial Sloan Kettering Cancer Center reported the highest average citations per paper.

**Table 2:** The top 10 productive institutions with publications.

Rank	Institutions	Countries	NP	NC	AC	H-index
1	Cornell University	USA	39	16,956	434.77	26
2	Sichuan University	China	37	1,005	27.16	18
3	Ruprecht Karls University Heidelberg	Germany	35	2,669	76.26	21
4	Helmholtz Association	Germany	34	2,265	66.62	21
5	University of California System	USA	31	9,132	294.58	22
6	Fudan University	China	30	2,236	74.53	19
7	Shanghai Jiao Tong University	China	28	1,680	60.00	18
8	Chinese Academy of Sciences	China	25	1,188	47.52	15
9	Memorial Sloan Kettering Cancer Center	USA	24	15,005	625.21	19
10	Chinese Academy of Medical Sciences Peking Union Medical College	China	24	2,861	119.21	16

**Figure 4:**
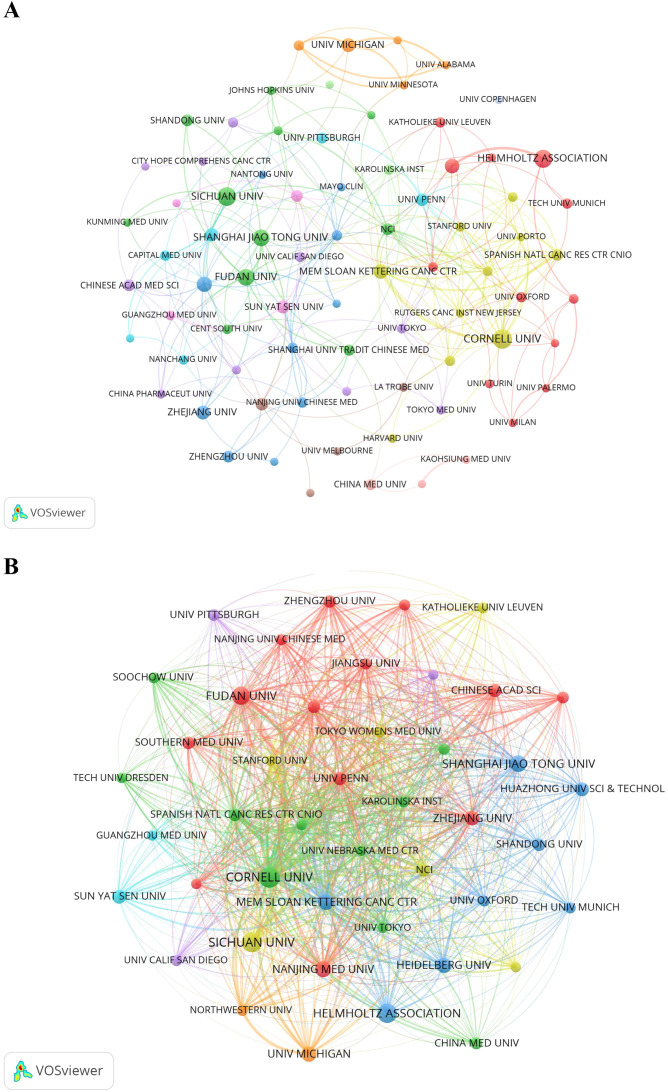
**(A)** The institution cooperation network visualization map generated by VOSviewer (version 1.6.20). **(B)** The institution citation network visualization map was generated by VOSviewer (version 1.6.20).

**Figure 4A** illustrates the collaboration network among the top 78 institutions, each with at least seven publications. These institutions were divided into 12 clusters based on their collaboration patterns, with closely linked institutions indicating active partnerships that drive the PMN field forward. **Figure 4B** presents a citation network map showing 44 items and 837 links. Cornell University had the highest TLS (1,942), followed by Memorial Sloan Kettering Cancer Center (TLS = 1,310) and the Spanish National Cancer Research Center (TLS = 954).

### 3.4 Authors and co-cited authors

A total of 7,955 authors contributed to the 1,303 publications in the PMN field. **Table 3** lists the top 10 most productive authors ranked by the number of publications. Six were from the United States, two from Japan, while Spain and Germany contributed one researcher each. David Lyden (Cornell University, USA) was the most prolific author, with 19 publications and the highest H-index (16), followed by Hector Peinado (Spanish National Cancer Research Center, Spain) and Lonnie D Shea (University of Michigan, USA). Irina Matei (USA) achieved the highest average citations per article, with an impressive 1,042.13.

**Table 3:** The top 10 productive authors with publications.

Rank	Author	NP	H-index	NC	AC	Countries	Institutions
1	Lyden, David	19	16	15,983	841.21	USA	Cornell University
2	Peinado, Hector	14	11	11,432	816.57	Spain	Spanish National Cancer Research Center
3	Shea, Lonnie D.	12	9	503	41.92	USA	University of Michigan
4	Ochiya, Takahiro	11	8	702	63.82	Japan	Tokyo Medical University
5	Krüger, Achim	10	9	510	51.00	Germany	Technical University of Munich
6	Bushnell, Grace G.	10	8	381	38.10	USA	University of Michigan
7	Maru, Yoshiro	10	6	332	33.20	Japan	Tokyo Women’s Medical University
8	Kang, Yibin	9	8	8,793	977.00	USA	Princeton University
9	Jeruss, Jacqueline S.	9	7	368	40.89	USA	University of Michigan
10	Matei, Irina	8	6	8,337	1042.13	USA	Cornell University

**Figure 5:**
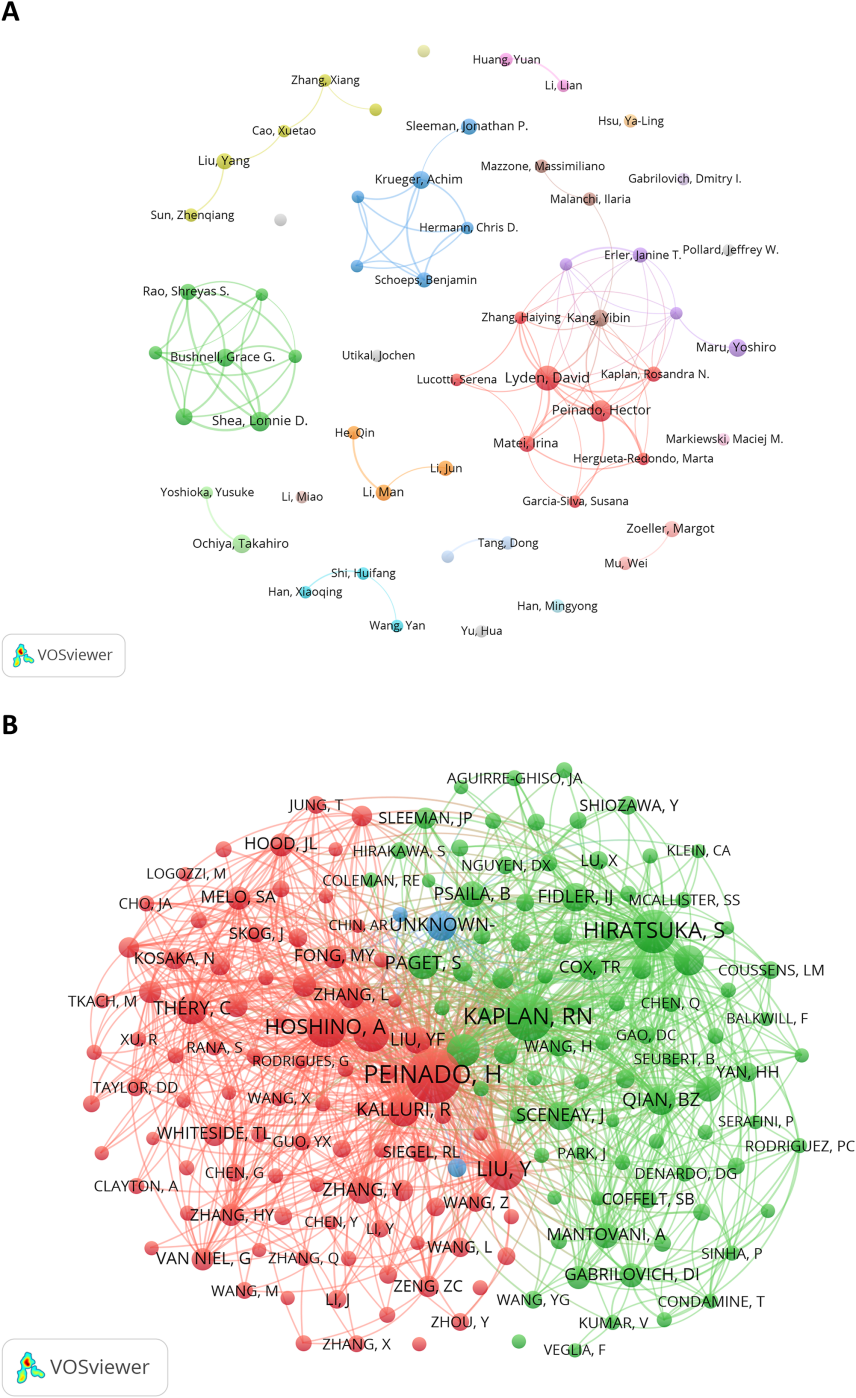
**(A)** The author cooperation network visualization map generated by VOSviewer (version 1.6.20). **(B)** The author co-citation network visualization map generated by VOS viewer (version 1.6.20).

**Figure 5A** illustrates the collaboration network of 57 authors who each published at least five articles. Prolific authors like David Lyden, Hector Peinado, Yibin Kang, Achim Krüger, and Grace G Bushnell had the active network of collaborators. The network also includes independent authors, such as Ya-Ling Hsu, Dmitry I Gabrilovich and Jochen Utikal. **Figure 5B** presents the co-citation network, which contains 184 items, 3 clusters, and 15,908 links. Hector Peinado (TLS = 14,853), Rosandra N Kaplan (TLS = 10,363), and Sachie Hiratsuka (TLS = 9,841) had the highest TLS, indicating their significant influence in the PMN field.

### 3.5 Journals and co-cited journals

Altogether, 400 journals published PMN-related articles, with journal IF and Journal Citation Report (JCR) quartile serving as key indicators of their influence. **Table 4** lists the top 10 journals by the number of publications, accounting for 25.7% (n=335) of all articles in the PMN field. Among these, three Swiss journals occupied the top three spots: *Cancers* (70 articles, IF 2023 = 4.5, Q1), *International Journal of Molecular Sciences* (60 articles, IF 2023 = 4.9, Q1/2), and *Frontiers in Oncology* (41 articles, IF 2023 = 3.5, Q2).

**Table 4:** The top 10 research journals by publication volume.

Rank	Journals	NP	Country	IF (2023)	JCR Quartile	H-index	Total citations
1	*Cancers*	65	Switzerland	4.5	Q1	22	1,312
2	*International Journal of Molecular Sciences*	60	Switzerland	4.9	Q1/2	24	1,857
3	*Frontiers In Oncology*	41	Switzerland	3.5	Q2	14	1,033
4	*Cancer Research*	40	USA	12.5	Q1	29	5,132
5	*Frontiers In Immunology*	30	Switzerland	5.7	Q1	14	1,670
6	*Cancer Letters*	22	Netherlands	9.1	Q1	16	894
7	*Scientific Reports*	21	United Kingdom	3.8	Q1	13	422
8	*Journal of Experimental & Clinical Cancer Research*	20	Italy	11.4	Q1	11	627
9	*Seminars In Cancer Biology*	19	United Kingdom	12.1	Q1	17	2,040
10	*Oncogene*	17	United Kingdom	6.9	Q1	16	1,167

**Supplementary Table 1** provides additional insights into the top 10 co-cited journals, ranked by co-citation frequency, IF (2023), and JCR quartile. The most frequently co-cited journals included *Cancer Research* (IF 2023 = 12.5, Q1, 5,889 co-citations) and *Nature* (IF 2023 = 50.5, Q1, 4,142 co-citations). Of the top 10 co-cited journals, nine were ranked in Q1 of the JCR, and seven had an IF exceeding 10. Notably, *Cancer Research* achieved the highest H-index (29) among all journals, along with the most citations (5,132) and co-citations (5,889), underscoring its significant impact in the field.

**Figure 6:**
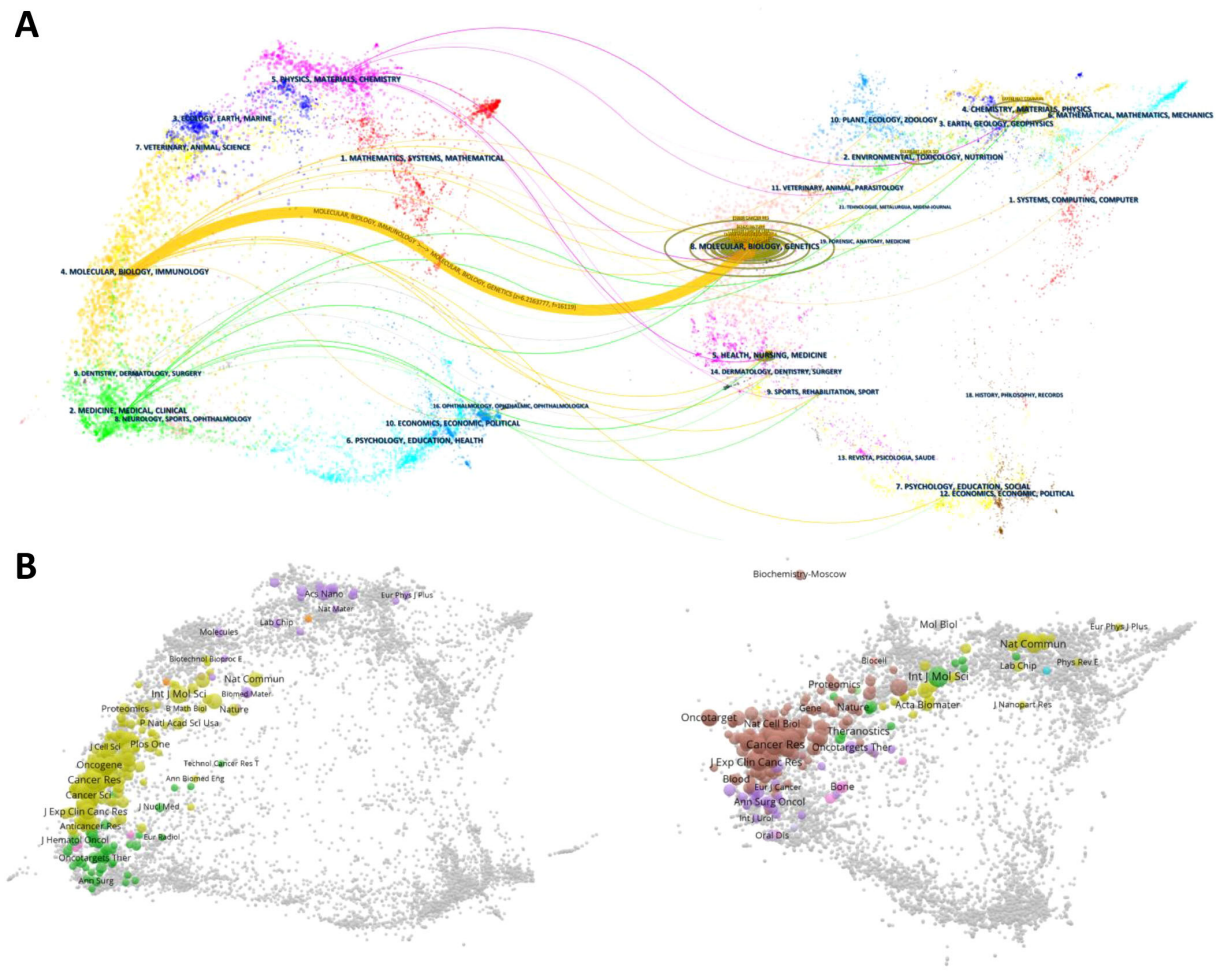
**(A)** presents a double-map overlay of citing and cited journals for the 1,303 PMN-related articles generated by CiteSpace (version 6.3.R3). This visualization maps citation relationships across related fields. The left side represents citing journals, which reflect applied research in PMN, while the right side represents cited journals, serving as the research foundation. Lines connecting the maps indicate citation paths across disciplines, with different colors representing subject areas and thicker lines denoting stronger links. **(B)** displays the overlay of citing and cited journals, where the dual maps can explore specific journal relationships and connections.

As shown in **Figure 6**, citing journals were predominantly spread across subjects #2 (Medicine, Medical, Clinical) and #4 (Molecular, Biology, Immunology), while cited journals were mainly centered in subject #8 (Molecular, Biology, Genetics). Subsets of cited journals also extended into subjects #2 (Environmental, Toxicology, Nutrition), #4 (Chemistry, Materials, Physics), and #5 (Health, Nursing, Medicine). The strongest connection, represented by the thickest orange line, highlights the significant influence of Molecular Biology and Genetics on Molecular Biology and Immunology (z = 6.22, f = 16119).

### 3.6 Cited and co-cited references

**Figure 7:**
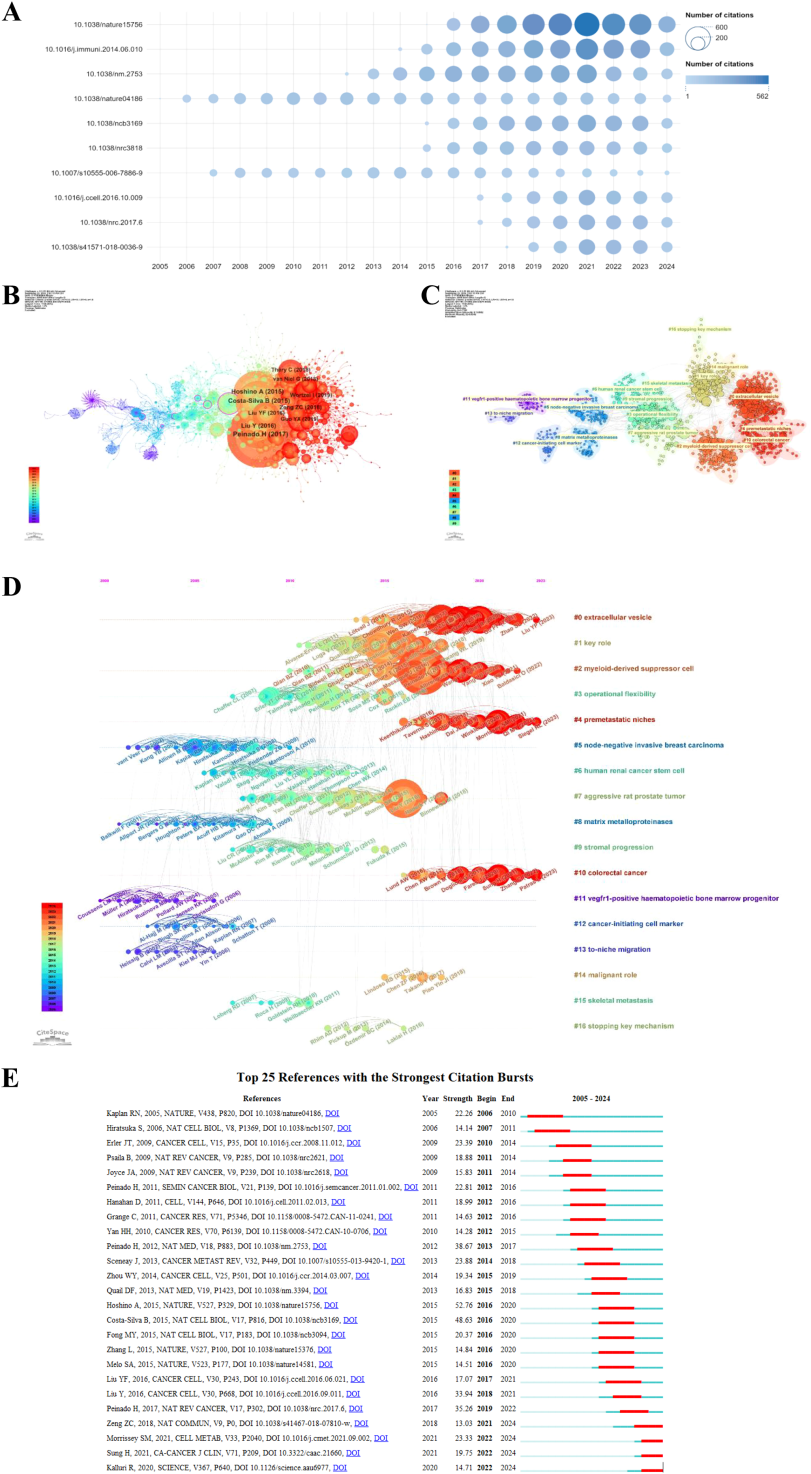
The top 10 most cited articles in the PMN field are shown in **(A)**, with bubble size representing citation frequency and colors ranging from light blue to dark blue to indicate increasing citation counts. DOI numbers replace article titles for clarity. **(B)** illustrates the co-citation network map of the top 10 references, generated using CiteSpace (version 6.3.R3), where node size corresponds to the number of co-citations, and labels include the first author and publication year. **(C)** presents the clustering network map of references, with clusters labeled using reference keywords and numbered ascendingly, such that lower numbers indicate clusters with more studies. **(D)** displays the timeline view of co-cited references, with nodes arranged by publication year; older references are on the left, and newer ones on the right. Nodes aligned horizontally represent clusters, with the duration of each cluster indicated by the length of its horizontal line. **(E)** highlights the top 25 references with the strongest citation bursts, where blue segments represent time intervals, and red segments indicate active periods of citation bursts, showcasing rapidly growing research areas in the PMN field.

The impact of an article in the PMN field is often reflected by its citation count. **Table 5** and **Figure 7A** highlight the top 10 most cited articles, with two published in *Nature* and 5 in *Nature*-affiliated journals. The most frequently cited article was “Tumor exosome integrins determine organotropic metastasis” (Ayuko Hoshino et al., 2015), with 3,473 citations. This groundbreaking study demonstrated how tumor exosome integrins establish PMN by interacting with organ-specific cells, paving the way for diagnostic and therapeutic innovations in organotropic metastasis (17).

**Table 5:** Top 10 highly cited references.

Rank	Title	Journal	First author	Year	NC	DOI
1	Tumour exosome integrins determine organotropic metastasis	*Nature*	Hoshino A	2015	3,473	10.1038/nature15756
2	Tumor-Associated Macrophages: From Mechanisms to Therapy	*Immunity*	Noy R	2014	2,863	10.1016/j.immuni.2014.06.010
3	Melanoma exosomes educate bone marrow progenitor cells toward a pro-metastatic phenotype through MET	*Nature Medicine*	Peinado H	2012	2,801	10.1038/nm.2753
4	VEGFR1-positive hematopoietic bone marrow progenitors initiate the pre-metastatic niche	*Nature*	Kaplan R N	2005	2,468	10.1038/nature04186
5	Pancreatic cancer exosomes initiate pre-metastatic niche formation in the liver	*Nature Cell Biology*	Costa-Silva B	2015	1,934	10.1038/ncb3169
6	Revisiting STAT3 signaling in cancer: new and unexpected biological functions	*Nature Reviews Cancer*	Yu H	2014	1,627	10.1038/nrc3818
7	Matrix metalloproteinases and tumor metastasis	*Cancer and Metastasis Reviews*	Deryugina E I	2006	1,626	10.1007/s10555-006-7886-9
8	Extracellular Vesicles in Cancer: Cell-to-Cell Mediators of Metastasis	*Cancer Cell*	Becker A	2016	1,206	10.1016/j.ccell.2016.10.009
9	Pre-metastatic niches: organ-specific homes for metastases	*Nature Reviews Cancer*	Peinado H	2017	1,204	10.1038/nrc.2017.6
10	Extracellular vesicles in cancer – implications for future improvements in cancer care	*Nature Reviews Clinical Oncology*	Xu R	2018	1,021	10.1038/s41571-018-0036-9

Using a scaling factor of k = 25, we retrieved 1,303 articles with the g-index and identified 1,180 cited references. Co-citation and cluster analysis revealed core knowledge and evolutionary hotspots in the PMN field. **Supplementary Table 2** lists the top 10 co-cited references, with the co-citation network visualized in **Figure 7B**. The most co-cited reference, “Pre-metastatic niches: organ-specific homes for metastases” (Hector Peinado et al., 2017), summarized key mechanisms of PMN formation and its novel processes.

The co-citation analysis identified 17 clusters, with modularity Q and mean profile S above 0.75, indicating strong clustering effects (**Figure 7C**). The timeline view (**Figure 7D**) illustrates the evolution of research hotspots over time. Early clusters, such as #5 (node-negative invasive breast carcinoma), #8 (matrix metalloproteinases), #11 (vegfr1-positive hematopoietic bone marrow progenitor), #12 (cancer-initiating cell marker), and #13 (to-niche migration), represent initial research topics in the PMN field. However, these hotspots have since shifted. Current clusters, such as #0 (extracellular vesicle), #2 (myeloid-derived suppressor cell), #4 (PMN), and #10 (colorectal cancer), reflect emerging areas of interest. Additionally, the research now encompasses key topics linked to other cancers, including breast cancer, pancreatic cancer, and hepatocellular carcinoma, highlighting the growing scope of PMN-related studies.

Citation burst analysis provides insight into rapidly growing research areas. The top 25 references with the highest citation bursts are presented in **Figure 7E**. The earliest burst, attributed to Rosandra N Kaplan et al.’s 2005 study, proposed the concept of PMN, demonstrating that bone marrow-derived hematopoietic progenitor cells create favorable microenvironments for tumor cells. This seminal work sparked interest in PMN interventions to prevent metastasis. The strongest citation burst (strength = 52.76) was observed in Ayuko Hoshino et al.’s 2015 study in Nature, which lasted until 2020. Notably, 2016 had the highest number of new citation bursts (5), followed by 2012 (4), indicating that high-burst papers drove significant research activity during these years. The most recent citation burst, observed in 2022 and still ongoing, is driven primarily by three influential articles: Samantha M Morrissey et al. (2021) in *Cell Metabolism* (18), Hyuna Sung et al. (2021) in *CA: a Cancer Journal for Clinicians* (19), and Raghu Kalluri et al. (2020) in *Science* (20). These results underscore the dynamic and evolving nature of research in the PMN field.

### 3.7 Keywords analysis of research hotspots

**Figure 8:**
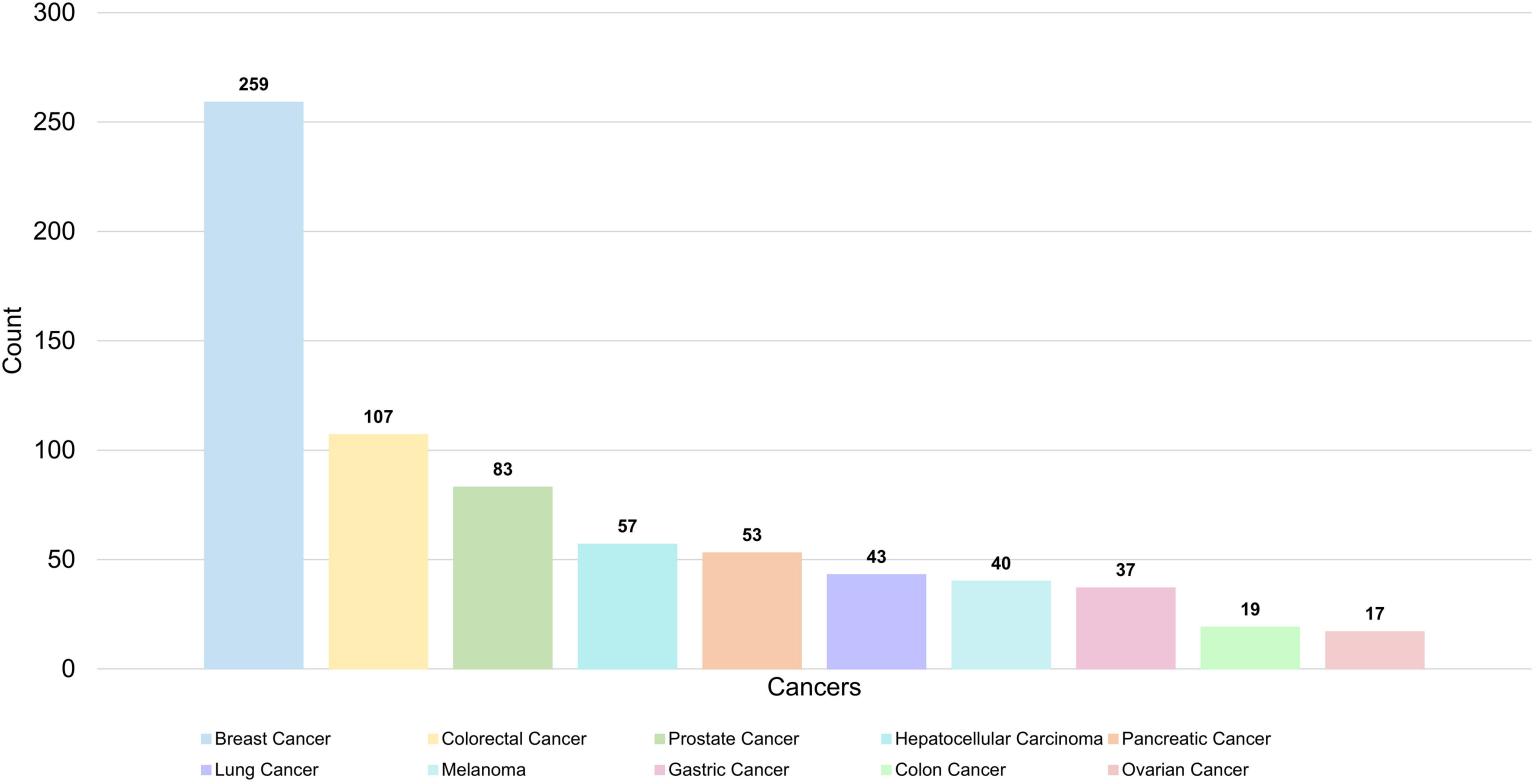
The top 10 most common cancers in PMN field.

A total of 336 keywords were identified from 1,303 articles in the PMN field. The most commonly used molecular keywords included exosomes (N = 319), EVs (N = 241), and microRNAs (N = 80), while stem cells (N = 118), mesenchymal stem cells (N = 71), and CTCs (N = 71) were the most frequently used cellular keywords. Pathological processes frequently referenced included metastasis (N = 395), expression (N = 238), and epithelial-mesenchymal transition (N = 156). Among cancers, the most studied were breast cancer (N = 259), colorectal cancer (N = 107), and prostate cancer (N = 83) (**Table 6**, **Figure 8**).

**Table 6:** The top 10 molecules, cells, pathological processes and cancers in PMN field.

Molecules	Count	Cells	Count	Pathological processes	Count	Cancers	Count
Exosomes	319	Stem Cells	118	Metastasis	395	Breast Cancer	259
Extracellular Vesicles	241	Mesenchymal Stem Cells	71	Expression	238	Colorectal Cancer	107
MicroRNAs	80	Circulating Tumor Cells	69	Epithelial-Mesenchymal Transition	156	Prostate Cancer	83
Extracellular Matrix	77	Tumor-Associated Macrophages	68	Angiogenesis	147	Hepatocellular Carcinoma	57
Microvesicles	68	Dendritic Cells	67	Growth	131	Pancreatic Cancer	53
Endothelial Growth Factor	56	Macrophages	66	Progression	114	Lung Cancer	43
Lysyl Oxidase	53	Myeloid Cells	64	Inflammation	99	Melanoma	40
TGF-β	53	T Cells	59	Activation	94	Gastric Cancer	37
Protein	48	Progenitor Cells	49	Promote	90	Colon Cancer	19
NF-κB	42	Cancer Cells	48	Invasion	70	Ovarian Cancer	17

**Figure 9:**
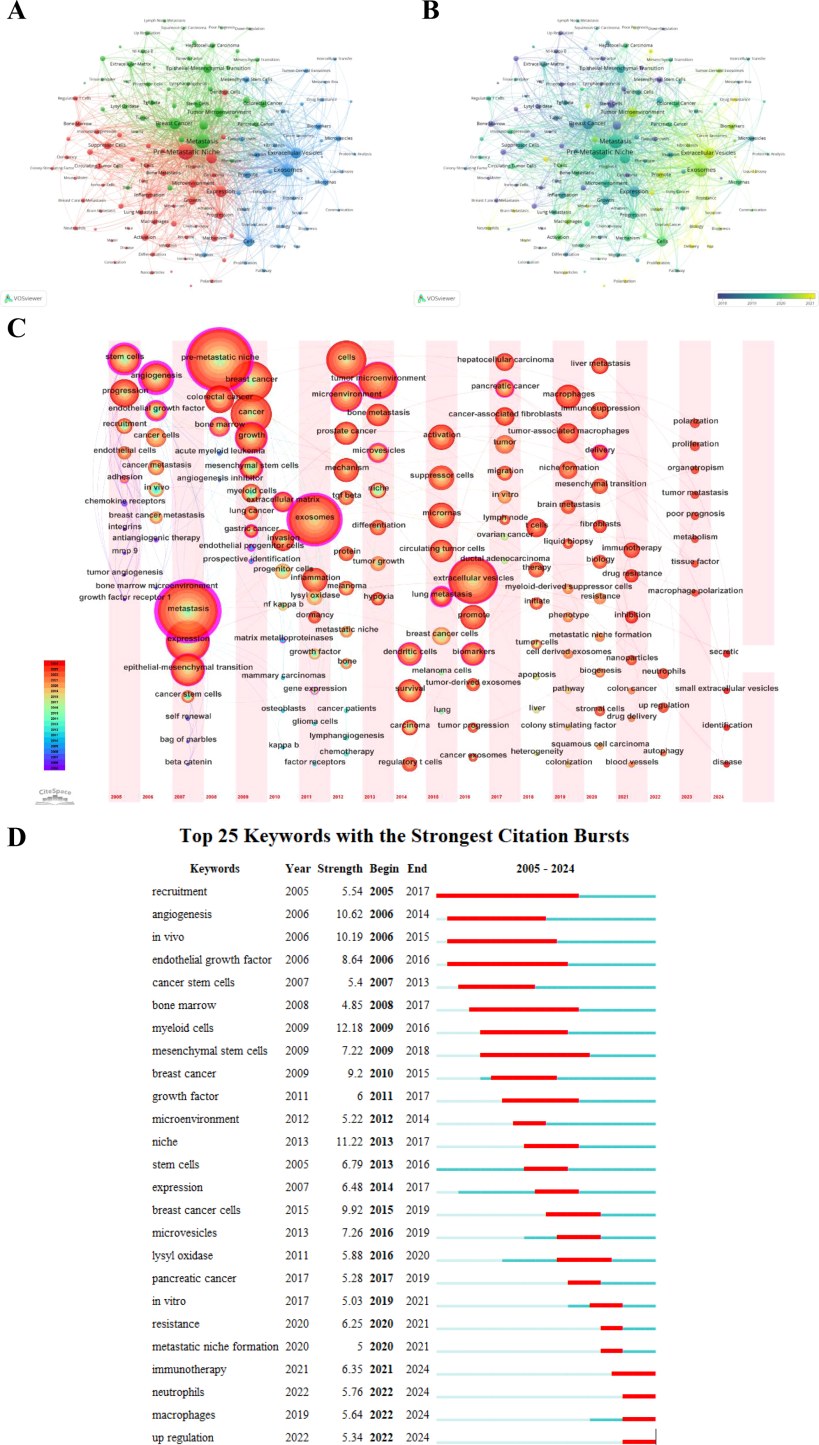
**(A)** shows the network visualization map of keywords generated by VOSviewer (version 1.6.20), highlighting clusters of interconnected terms that represent distinct research areas. **(B)** illustrates the evolution of keywords over time, where nodes are colored based on their average appearance year, with earlier terms in purple and more recent ones in yellow. **(C)** provides a time zone view of keywords generated by CiteSpace (version 6.3.R3), displaying the chronological emergence of key terms and their contribution to research evolution. **(D)** highlights the top 25 keywords with the strongest citation bursts, where blue line segments represent time intervals, and red segments indicate periods of heightened activity. These bursts reflect rapidly growing interest in specific topics and emerging hotspots in PMN research.

We performed a cluster analysis of keywords to explore the distribution of topics within the PMN research field. **Figure 9A** shows a keyword network map revealing three distinct clusters. The central red cluster focuses on the study of PMN and organotropic metastases, their relationship with pathological processes in the microenvironment, and the role of associated cells, including bone metastasis, lung metastasis, brain metastasis, growth, progression, inflammation, immunosuppression, circulating tumor cells, and immune cells (e.g., macrophages, neutrophils, T cells, dendritic cells). The green cluster highlights PMN associations with specific diseases, pathological processes of tumor metastasis, and related molecular proteins, including breast cancer, colorectal cancer, pancreatic cancer, metastasis, tumor microenvironment, epithelial-mesenchymal transition, transforming growth factor-β (TGF-β), vascular endothelial growth factor (VEGF), and nuclear factor kappa-B (NF-κB). The blue cluster centers on EVs and their connection to PMN, along with their diagnostic, therapeutic, and prognostic roles in tumor metastasis, encompassing terms like exosomes, microvesicles, intercellular transfer, communication, microRNAs, biomarkers, liquid biopsy, proteomic analysis, and delivery.

We mapped keywords to a timeline using VOSviewer (version 1.6.20) and CiteSpace (version 6.3.R3), resulting in two time-varying keyword visualization maps. **Figure 9B** illustrates the evolution of PMN research clusters over time. Purple nodes represent keywords that first emerged, on average, before 2018, while yellow nodes indicate keywords that have gained prominence in recent years, particularly after 2021. **Figure 9C**, a keyword timezone map, shows the chronological progression of research hotspots. Between 2005 and 2024, research trends have shifted from stem cell and bone marrow progenitor cell studies and TDSFs to EVs and immunotherapy, reflecting the dynamic evolution of the field.

Citation bursts provide another perspective on academic hotspots and emerging research trends. **Figure 9D** displays the top 25 keywords with the highest citation burst intensities. Notable examples include “myeloid cells” (12.18), “niche” (11.22), “angiogenesis” (10.62), “*in vivo*” (10.19), and “breast cancer cells” (9.92). Recent bursts observed in 2024 for keywords such as immunotherapy, neutrophils, macrophages, and upregulation highlight growing areas of interest that may define future PMN research directions.

## 4 Discussion

Bibliometric maps and visualizations of 1,303 PMN-related publications extracted from the WOSCC database (2005–2024) were analyzed using more than five advanced scientific tools. This study systematically evaluated the current state of PMN research and identified promising future directions in the field.

### 4.1 General distribution

This study analyzed 1,303 PMN-related articles published between January 1, 2005, and September 18, 2024, sourced from 1,627 institutions and authored by 7,955 researchers. The data reveal the increasing global attention to PMN research. Since Rosandra N Kaplan et al. introduced the concept of the “pre-metastatic niche” in *Nature* in 2005, the field has experienced significant growth. By 2023, the NP was approximately five times that of 2013, and the total NC had increased tenfold over the same period, underscoring rapid advancements in this area (**Figure 2**).

China and the United States were the leading contributors to PMN research. China ranked first in publication output, accounting for 34.1% of the total (444 articles), while the United States ranked second with 29.5% (385 articles). However, the United States led in the NC (48,655) and H-index (95), highlighting the superior quality and impact of its research. Spain’s articles had the highest average citations per article (229.91). The USA-China collaboration was the most prominent, with other countries, including Germany, the United Kingdom, Spain, Italy, and Japan, also playing significant roles in advancing the PMN field and fostering international partnerships.

Institutional contributions reflected these national trends. Among the top ten institutions, five were based in China, three in the United States, and two in Germany. United States institutions, including Cornell University, the University of California System, and Memorial Sloan Kettering Cancer Center, demonstrated the highest citation counts and H-index values. These results underscore the pivotal role of first-class colleges and research institutions in elevating a country’s academic standing and global influence.

Six of the ten most productive scientists were from the United States, two were from Japan, while Spain and Germany contributed one researcher each. David Lyden of Cornell University pioneered the PMN field, introducing the concept in 2005 and consistently producing influential research. His foundational study, “VEGFR1-positive hematopoietic bone marrow progenitors initiate the pre-metastatic niche,” remains a cornerstone of PMN research. For nearly 20 years, his research has driven key advancements and breakthroughs in the PMN field, serving as a foundational reference for other scientists. Other prominent contributors include Hector Peinado from the Spanish National Cancer Research Center, Lonnie D Shea from the University of Michigan, and Rosandra N Kaplan from the National Institutes of Health. These researchers, along with their extensive network of collaborators, have significantly shaped the PMN research landscape (**Figure 5**).

These individuals and their groups were more likely to publish impactful research on PMN. While China ranked first in the NP and demonstrated extensive international collaboration, the data suggest a relative lack of high-level scholars and research teams in comparison to other leading countries.

Key journals such as *Cancers*, *International Journal of Molecular Sciences*, *Frontiers in Oncology*, *Cancer Research*, and *Frontiers in Immunology* have been instrumental in disseminating PMN-related findings. *Cancers* published the highest number of PMN-related articles, while *Cancer Research* had the highest total citations and co-citations, indicating that these journals are well-positioned to continue publishing significant discoveries in PMN research. High-impact journals such as *Nature*, *Nature Reviews Cancer*, *Cancer Cell*, and *Cell* (all JCR Q1) further underscore the PMN field’s significance in tumor metastasis research (**Supplementary Table 1**).

The dual-map overlay analysis (**Figure 6**) highlights that PMN field studies are primarily published in molecular biology, immunology, and clinical medicine journals. Advances in foundational sciences (such as molecular biology, genetics, chemistry, materials, physics, etc.) continue to provide critical support for understanding PMN mechanisms and translating findings into clinical applications. The multidisciplinary collaboration between these fields is poised to accelerate research progress and deliver impactful patient outcomes.

### 4.2 Hotspots and frontiers

Co-citation analysis of references and keyword co-occurrence networks provide a comprehensive overview of the structure of PMN research. Tools like timeline views of cited references and timezone maps of publication keywords illustrate the dynamic evolution of research hotspots in the PMN field. This analysis reveals key focus areas, including metastatic organotropism, extracellular vesicles, innate immunocytes (e.g., macrophages and neutrophils), and immunotherapy. These insights highlight current research priorities and emerging academic frontiers. The following subsections delve deeper into these areas, exploring their significance for the field and their potential to shape future research.

#### 4.2.1 Metastatic organotropism

The ability of certain tumors to spread and invade specific distant organs, including the liver, lung, bone, and brain, is known as metastatic organotropism (21). Metastatic organotropism is among PMN’s defining characteristics (10). Studies have shown that PMN composition in particular organs significantly influences organ-specific metastatic spread and determines cancer cell organotropism (6, 22). Bone, lung, liver, brain, and lymph node metastases are the most common organotropic metastases in PMN research, as shown in **Table 6** and **Figure 9**. PMN characteristics are shared across different organs; however, unique cell types and microenvironments result in some organ-specific PMN traits. For example, bone metastasis is common in patients with lung, breast, and prostate cancer; the formation of bone PMNs is driven by osteoclast and osteoblast activation, along with angiogenesis, to provide a growth-permissive environment for CTCs (23). Lung metastasis frequently occurs in patients with breast cancer, upper gastrointestinal cancer, and renal cell carcinoma; the formation of lung PMNs recruits bone marrow-derived cells to support inflammation and immunosuppression, and this state is exacerbated by reprogrammed lung fibroblasts, alveolar epithelial cells, and macrophages (24). Liver metastasis is commonly seen in patients with pancreatic, colorectal, and gastric cancers, among others. Liver PMNs show the influx of bone marrow-derived cells, fibrosis, and immunosuppression in which hepatic stellate and Kupffer cells play an essential role (25). Brain metastasis is common in patients with lung cancer, breast cancer, and melanoma. Brain PMNs have higher blood-brain barrier permeability and local immunosuppression, and are rich in neurons and glial cells, providing a suitable environment for the growth of CTCs (26). Lymph node PMNs exhibit lymphangiogenesis and remodeling of high endothelial venules, facilitating tumor dissemination through the lymphatic system (27).

The connection between PMN and metastatic organotropism is attracting increasing attention. Early studies mainly focused on how TDSFs influence adhesion molecules and ECM in distal organs to promote organotropic metastasis (28, 29). More recent researches, however, have identified tumor-derived EVs as pivotal factors in determining metastatic organotropism (30–33).

Exosomes, a subset of EVs approximately 30–150 nm in diameter (34), play a critical role in this process. Ayuko Hoshino et al. were the first to demonstrate the decisive role of tumor exosome integrins (ITGs) in organotropic metastasis (17). Their findings showed that the specific fusion of organ-resident cells with tumor exosome ITGs activates Src phosphorylation and upregulates pro-inflammatory S100 expression, promoting PMN formation and organ-specific metastasis. Notably, specific associations have been identified: exosomal ITGs α_6_β_4_ and α_6_β_1_ predominantly target lung tissues, facilitating lung metastases, while ITGs α_v_β_5_ demonstrate a similar role in liver metastases. The relationship between exosomal ITGs and metastases in lymph nodes (35), bone (36), and brain (37) has also been reported. Another critical facet of metastatic organotropism is that tumor-derived EVs are taken up by specific cell types in individual organs, creating a microenvironment conducive to metastasis (38). For example, fibroblasts (39) and epithelial cells (40) in the lung internalize exosomes from lung-tropic tumor cells, Kupffer cells (32) in the liver internalize exosomes from liver-tropic tumors, and microglia and brain endothelial cells take up exosomes from brain-tropic tumors (41).

The VEGF, TGF-β, and C-X-C chemokine ligand 12 (CXCL12)/C-X-C chemokine receptor type 4 (CXCR4) signaling pathways are pivotal in PMN formation and organotropic metastasis. The VEGF signaling primarily regulates vascular endothelial cell proliferation and migration, promotes angiogenesis in PMNs, increases vascular permeability, and facilitates CTC invasion and metastasis. Wei Mu et al. demonstrated that benzo[a]pyrene exposure induces hepatocellular carcinoma exosome-derived circular RNA, which activates VEGF expression in lung fibroblasts, promoting lung metastasis (42). The TGF-β pathway controls critical cellular processes such as migration, invasion, ECM remodeling, and immunosuppression. Studies by Anyi Liu et al. (43), Fangting Liu et al. (44), and Xing-Ning Lai et al. (45) indicate that aberrant TGF-β activation enhances tissue-specific metastasis by upregulating organ-specific metastasis-related genes. The CXCL12-CXCR4 axis is a critical mediator of metastasis, recruiting CTCs and immune cells within the PMN (46). Zhen Wang et al. reported that CD62L^dim^ neutrophils aggregate in the pre-metastatic lung microenvironment via the CXCL12-CXCR4 pathway, promoting lung metastasis in breast cancer (47).

Targeting these PMN-promoting pathways can inhibit PMN formation and reduce organotropic metastasis risk. The TGF-β inhibitors, such as monoclonal antibodies or soluble TGF-β receptors, block TGF-β signaling (48), and have shown promising results in preclinical and early clinical trials (49). The VEGF inhibitors, including anti-VEGF drugs such as bevacizumab and sunitinib, and VEGF receptor (VEGFR) inhibitors such as sorafenib, suppress tumor angiogenesis, growth, and PMN formation by blocking VEGF-A binding to VEGFR and subsequent VEGF signal transduction (50). NOX-A12, a polyethylene glycolated mirror oligonucleotide that binds CXCL12 to inhibit its interaction with CXCR4. Meggy Suarez-Carmona et al. reported its high safety and tolerability in patients with advanced metastatic colorectal and pancreatic cancer (51). The CXCR4-targeting therapies include non-peptide antagonists (such as AMD3100) and peptide-based antagonists (such as PL-Peptide R) (52). AMD3100 (Plerixafor) is the only FDA-approved CXCR4 antagonist. Jian Jiang et al. demonstrated that combining AMD3100 with combretastatin A4 nanodrug, a neovascularization disruptor, significantly inhibited tumor growth and lung metastasis in mice with breast cancer (53). Moreover, Caterina Ieranò et al. developed PL-Peptide R, a peptide R-modified stealth liposome, which inhibited CXCR4-dependent migration *in vitro*, reduced lung metastasis, and improved survival in melanoma-affected mice (54).

Despite these advancements, several key questions remain, including the impact of tumor heterogeneity on organ-specific PMN formation, the regulatory mechanisms of ECM remodeling in PMN, and the influence of PMN evolution over time on organotropic metastasis. Further exploration of these areas is essential. Emerging advances in science and technology have greatly facilitated the systematic study of PMN’s key biological characteristics and organotropism. First, developments in materials technology have enabled researchers to biologically engineer 3D models mimicking the complexity of tissues (55–57). For instance, Ryan A Carpenter et al. seeded human bone marrow stromal cells onto a hydrogel scaffold, which was then implanted subcutaneously in mice to induce vascularization and a pro-inflammatory microenvironment, forming a tissue-engineered PMN that subsequently recruited CTCs from orthotopic prostate tumor xenografts (58). These bioengineered PMNs have significantly advanced our understanding of PMN development and metastatic organotropism, while also facilitating the testing of anti-tumor therapies in high-throughput settings (59). Second, the advent of spatial transcriptomics, single-cell sequencing, advanced imaging techniques, and artificial intelligence has enabled researchers to analyze PMN components comprehensively and predict metastatic organotropism (60). For example, Joseph J Zhao et al. revealed spatially resolved ecological niches and altered tumor microenvironments associated with peritoneal metastasis in gastric cancer (61). Linda Bojmar et al. developed a pre-metastatic liver multiparameter profile capable of predicting early metastasis outcomes after pancreatic cancer surgery with an accuracy of 78% (8). Third, it is important to highlight that microphysiological systems (MPS) have emerged as powerful tools in organotropic metastasis research. These systems encompass 3D cell co-culture platforms and organ-on-a-chip models that integrate microfluidics, biology, and engineering to replicate complex organ-specific microenvironments *in vitro* (62). The MPS enables real-time monitoring, morphological analysis, and protein quantification, enhancing our understanding of tumor migration, angiogenesis, and PMN formation (63).

#### 4.2.2 Extracellular vesicles

EVs are heterogeneous lipid bilayer membrane structures secreted by cells (64) and are commonly found in human body fluids such as blood, urine, and saliva. Based on their size and biogenic mechanism, EVs can be classified into exosomes, microvesicles, apoptotic bodies, and oncosomes (64, 65). Over the past decade, evidence has increasingly pointed to EVs as essential mediators of cancer progression and metastasis through cell-cell communication (66–68). Tumor-derived EVs (TDEs) are the primary focus in PMN research, as their cargo—comprising proteins, mRNAs, miRNAs, small RNAs, DNA fragments, lipids, and metabolites—has been shown to alter the host microenvironment and promote PMN formation, driving organ-specific metastasis (13, 25, 69, 70).

TDEs mediate PMN formation through various mechanisms, including promoting inflammation (71, 72), increasing angiogenesis and vascular permeability (73, 74), stimulating lymphangiogenesis (75), reprogramming stromal cells (76, 77), remodeling the ECM (78, 79), inducing immunosuppression (9, 80), and altering the metabolic milieu (81, 82). Recognizing their pivotal role, researchers have developed targeted strategies to block TDE-mediated metastasis, including inhibiting TDEs secretion (83), removing or destroying circulating TDEs (84), blocking TDEs interactions with recipient cells (85), and using TDEs as biomolecule or drug delivery tools to inhibit metastasis (86). For example, Niamh McNamee et al. demonstrated that calpeptin, Y27632, manumycin A, GW4869, and their combinations inhibited EV release from triple-negative breast cancer cells in *in vitro* studies (87). Eun-Ju Im et al. identified the oral antibiotic sulfisoxazole as an EV secretion inhibitor in breast cancer cells through screening and validation in animal models (88). Qunfei Tai et al. developed an antibody-functionalized capillary device for *in situ* exosome capture and quantification, successfully trapping Michigan Cancer Foundation-7 (MCF-7)-derived exosomes and inhibiting epithelial-mesenchymal transition in MCF-10A epithelial cells *in vitro* (89). Emma Rigg et al. reported that miR-146a-5p was highly expressed in brain tissue of patients with melanoma brain metastases and in melanoma cell-derived EVs, where it stimulated pro-tumorigenic cytokine secretion by cerebral astrocytes, promoting tumor progression. Deserpidine suppressed miR-146a-5p expression in EVs in both *in vivo* and *in vitro* experiments, reducing the incidence of melanoma brain metastasis (90). Moreover, as natural carriers, exosomes can be engineered as drug delivery vehicles, transporting exogenous RNAs, such as small interfering RNAs (siRNAs) and microRNAs, to target tissues or cells *in vivo*, modulating gene expression and inhibiting tumor metastasis (91). Liuwan Zhao et al. demonstrated in animal models that exosome membrane-encapsulated biomimetic nanoparticles (CBSA/siS100A4@Exosome) effectively delivered therapeutic siS100A4 to the lungs of mice, inhibiting lung metastasis post-breast cancer surgery (92). Sushrut Kamerkar et al. at The University of Texas MD Anderson Cancer Center engineered exosomes carrying siRNA targeting the KRAS^G12D^ oncogene, significantly suppressing pancreatic cancer progression and metastasis, thereby improving survival in mice (93). Based on these findings, MD Anderson Cancer Center is conducting a Phase I clinical trial (NCT03608631) to evaluate the safety and efficacy of EVs loaded with KRAS^G12D^-targeted siRNA in patients with metastatic pancreatic cancer.

However, clinical applications of TDE-targeted therapies still face challenges such as potential interference with normal cell functions, technical difficulties in isolating circulating TDEs, the possibility of immune responses triggered by TDEs destruction, and the need for large-scale production of stable, bioavailable TDEs carriers (94). Despite these obstacles, researchers agree that targeting TDEs is a promising avenue for inhibiting PMN formation and tumor metastasis (95).

In parallel, advances in liquid biopsy technology have highlighted TDEs as biomarkers for early detection, metastasis prediction, and tumor prognosis (96). Liquid biopsy leverages humoral fluids to detect tumor-related information, predict treatment responses, and guide the selection of personalized treatment options (97). Current biomarker research focuses on miRNAs and proteins within TDEs (70). For example, studies have shown that upregulated miR-223-3p, miR-92a-3p, miR-21-5p, and miR-342-3p in exosomes from gastric cancer ascites are associated with peritoneal metastasis (98). Similarly, high expression of cadherin 11 and integrin α5 in blood exosomes from breast cancer models correlated with bone metastasis (99). Lymph node metastasis is a strong prognostic indicator of poor outcomes in intrahepatic cholangiocarcinoma. A Phase I clinical trial (NCT06381648), initiated by Kyushu University and other universities in Japan, is actively recruiting patients to evaluate the use of exosomal biomarkers in liquid biopsy for preoperative detection of lymph node metastasis, aiding in treatment decision-making.

However, the lack of robust methods for isolating and analyzing TDE components limits further clinical application of liquid biopsy (100). As detection techniques improve and our understanding of TDE biology deepens, liquid biopsy is expected to play an increasingly significant role in PMN and metastasis research.

#### 4.2.3 Innate immunocytes

The coexistence of inflammation and immunosuppression is a hallmark of PMN and a major driver of cancer progression and metastasis (10). Innate immunocytes play a crucial role in mediating PMN-related immunosuppression and inflammation (9). Among them, macrophages and neutrophils have garnered significant research attention in recent years due to their pivotal functions in tumor metastasis.

##### 4.2.3.1 Macrophages

Macrophages are mononuclear phagocytic cells derived from monocytes (101). In PMN, the macrophage population includes bone marrow-derived macrophages (BMDMs) and tissue-resident macrophages, such as liver Kupffer cells, lung interstitial and alveolar macrophages, and brain microglia (102). TDSFs and TDEs secreted by primary tumors recruit BMDMs to future metastatic organs. These factors foster an inflammatory microenvironment, inducing the accumulation and proliferation of additional BMDMs (103, 104).

Furthermore, the macrophage phenotype is regulated by TDSFs and TDEs. Macrophages exhibit different functional subsets, including anti-tumor (M1 phenotype) and pro-tumor (M2 phenotype) subsets, which exert opposing effects (105). Current research focuses on the regulation of tissue-resident macrophages phenotypes and their roles in PMN formation and metastasis. For instance, Samantha M Morrissey et al. demonstrated that exosomes shed by lung cancer cells polarize alveolar interstitial macrophages into an immunosuppressive phenotype via NF-κB-dependent glycolysis-dominant metabolic reprogramming pathways. This process upregulates programmed cell death ligand 1 (PD-L1) expression and excretes large amounts of lactic acid, thereby creating an immunosuppressive niche that promotes tumor metastasis (18). Similarly, Hao Sun et al. observed that exosomes with high expression of miR-135a-5p are phagocytized by liver Kupffer cells. These exosomes promote the production of matrix metalloproteinase 7 and related signaling, decrease CD4^+^ T cells in the liver, and facilitate hepatic PMN formation (106). In summary, macrophages are influenced by their local microenvironment and exhibit remarkable functional plasticity. By fostering tumor-associated inflammation, remodeling the extracellular matrix, and inducing immunosuppression, they play pivotal roles in PMN formation and ultimately contribute to metastasis.

##### 4.2.3.2 Neutrophils

Neutrophils are the predominant component of peripheral white blood cells and are closely associated with inflammation and immune function (107). Advances in high-throughput sequencing have revealed the significant accumulation and critical functions of neutrophils in PMN. Neutrophil heterogeneity and neutrophil extracellular traps (NETs) are emerging as key research areas in the PMN field.

Neutrophils in PMN exhibit two phenotypic states, N1 (anti-tumor) and N2 (pro-tumor), influenced by the complex surrounding microenvironment (108, 109). Studies by Yanfang Liu et al. and Ching-Fang Wu et al. revealed that the lack of type I interferon in PMN, combined with abundant granulocyte colony-stimulating factor (G-CSF), IL-6, and IL-10 secretion by tumors, promotes N2 polarization (40, 110). These N2 neutrophils contribute to oxidative stress at the site of inflammation through the secretion of cytokines, such as tumor necrosis factor α, shaping the PMN’s inflammatory response, activating endothelial and stromal cells, and increasing circulating tumor cell adhesion (111, 112). Furthermore, N2 neutrophils suppress immune responses by mechanisms such as secreting type 1 arginase (ARG1) to deplete arginine, impairing T cell function, as demonstrated by Huajia Zhang et al. (113), and increasing PD-L1 expression under GM-CSF stimulation to inhibit T cell activation (114).

NETs, reticular structures composed of DNA, histones, and granular proteins, are released by neutrophils in response to stimuli (115). Evidence strongly links NETs to metastasis (116). Inflammatory factors like IL-8 and high mobility group box protein 1 in PMN, along with tumor-derived G-CSF, promote NETs formation (117). Mechanistic studies show that NETs contribute to metastasis in two main ways. First, WonJae Lee et al., Luyu Yang et al., and Álvaro Teijeira et al. demonstrated that NETs attract and capture CTCs through β-integrin-mediated adhesion and shield them from immune attack by cytolytic T lymphocytes and natural killer cells, facilitating colonization in PMN (118–120). Second, NET components indirectly influence tumor cell metastasis. Linbin Yang et al. found that NET DNA binds to transmembrane protein CCDC25 on breast cancer cells, enhancing motility (121). Additionally, Hamza O Yazdani et al. showed that neutrophil elastase activates the toll-like receptor-4 signaling pathway in colon cancer cells, promoting mitochondrial biogenesis and tumor growth (122). PD-L1 on NETs also contributes to immune evasion by depleting and impairing CD4^+^ and CD8^+^ T cells, as observed by Christof Kaltenmeier et al. (123).

#### 4.2.4 Immunotherapy

Immune checkpoint inhibitors (ICIs) are among the most successful types of immunotherapies and represent a prominent treatment for metastatic cancer. They target T cell checkpoints such as PD-L1, programmed cell death protein 1, and cytotoxic T lymphocyte antigen 4. However, the therapeutic success of ICIs has been limited to cancers with high mutation rates and is further hampered by the emergence of treatment resistance (124). Currently, no tailored therapies exist for preventing PMN formation, but innovative immune-based approaches are undergoing investigation and validation (9, 125).

The rapid development of advanced technologies, including single-cell sequencing, liquid biopsy, spatiotemporal omics, nanotechnology, and artificial intelligence, provides scientists with deeper insights into the immune landscape of PMN. These technologies facilitate the identification of immune determinants that drive the temporal and spatial evolution of PMN (9, 126–128). In the near future, these advancements are anticipated to inspire the development of next-generation immunotherapies that target critical immune cells involved in PMN formation. Such therapies aim to disrupt immunosuppressive environments within PMN and activate early anti-tumor immune responses, offering more effective strategies to combat metastasis (125).

##### 4.2.4.1 Targeting innate immunocytes

Blocking the recruitment of innate immunocytes like macrophages, neutrophils, and myeloid-derived suppressor cells (MDSCs) to PMN, inducing their depletion, or altering their immunosuppressive activity by reprogramming are currently common targeting strategies (129). Research to block macrophage recruitment is focused on inhibiting the cyclooxygenase-1/thromboxane A2 (130) and colony-stimulating factor-1/colony-stimulating factor-1 receptor (131) pathways and targeting the CXCL12/CXCR4 (132) and angiopoietin-2/TIE2 (133) axes. Methods to induce macrophage depletion include the use of liposomal clodronate (134) and trabectedin (135). CD40 agonists in combination with CSF-1R-targeting drugs (136), selective class IIa histone deacetylase inhibitors (137), and toll-like receptor agonists (138) can reprogram macrophages to enhance anti-tumor immunity.

Regarding neutrophil-targeted therapies, Zhiyuan Zheng et al. demonstrated that complement component C3 promotes neutrophil recruitment and NET formation, and that targeting the Th2 cell cytokine-STAT6-C3-NET axis can block neutrophil recruitment and NET formation in PMN (139). Other strategies to inhibit NET formation include blocking IL-17 (140), targeting tissue C5a receptor-1 and eliminating C5a (141), and using PAD4 drug inhibitors (118). Furthermore, Abhishek Tyagi et al. revealed that low concentrations of salidroside can inhibit N2 phenotype polarization of neutrophils in PMN and promote N1 phenotype polarization (142).

MDSCs, immature myeloid cells derived from the bone marrow, have immunosuppressive functions and are involved in multiple mechanisms of PMN generation. They represent a recent hotspot in immunotherapy research (143). Studies have shown that CXCR2 antagonists (144) and C‐C motif chemokine 2 or C‐C motif chemokine 2 receptor inhibitors (145, 146) can block MDSCs recruitment to PMN, and their combination with ICIs can improve the efficacy of immunotherapy. Research by Zhihao Lu et al. demonstrated that low-dose adjuvant epigenetic therapy can block MDSCs recruitment and promote their differentiation into a mesenchymal macrophage phenotype, disrupting the generation of postoperative lung PMN (147). Additionally, Traditional Chinese Medicine has shown beneficial effects in inhibiting MDSCs recruitment and tumor metastasis (148, 149).

Weakening the immunosuppressive function of MDSCs is another important approach in immunotherapy. Phosphodiesterase 5 inhibitors, including tadalafil and sildenafil, can downregulate ARG1 and inducible nitric oxide synthase expression, reverse MDSCs-induced immunosuppression, enhance anti-tumor immunity, and reduce the incidence of postoperative metastasis (150, 151).

##### 4.2.4.2 Targeting adaptive immunocytes

The immune surveillance roles of T-cells and B-cells, categorized as adaptive immunocytes, are frequently suppressed by innate immunocytes within PMN. This suppression is driven by TDSFs and EVs, leading to a reduction in cytotoxic and effector T cells and an expansion of regulatory T cells and regulatory B cells, thereby exacerbating PMN immunosuppression and promoting metastasis (152, 153).

Sabina Kaczanowska et al. utilized genetically engineered myeloid cells to deliver IL-12, inducing adaptive immune responses in tumor-bearing mice. Their findings demonstrated antigen presentation and T-cell activation, which reversed pulmonary PMN immunosuppression and significantly reduced lung metastasis incidence (154).

Additionally, the metastatic S100A4 protein facilitates T cell recruitment to the lung PMN, inducing a Th1/Th2 imbalance that shifts toward a pro-tumor Th2 phenotype. In a study involving mice with spontaneous breast cancer, Birgitte Grum-Schwensen et al. reported that the 6B12 antibody effectively blocked S100A4 activity, restored T cell polarization balance, and inhibited lung metastasis (155).

Regarding B cell research, Yan Gu et al. observed that tumor-domesticated B cells aggregate within lymph nodes and secrete pathological antibodies targeting the glycosylated membrane protein HSPA4. This interaction facilitates lymph node PMN formation and promotes breast cancer metastasis by activating the CXCR4/SDF1α axis (156). However, immunotherapy strategies targeting HSPA4 remain in the early stages and require further investigation.

##### 4.2.4.3 Nanotechnology-enhanced immunotherapies

To date, nanotechnology has demonstrated significant potential in enhancing immunotherapy for metastatic cancer, offering promising avenues for disrupting the biological processes underlying tumor metastasis (157). Strategically designed nanomaterials can inhibit metastasis-related immunosuppressive cells, reshape the immune microenvironment of the PMN, and reduce the likelihood of tumor metastasis (158). Several successful attempts have already been reported. For instance, Weiya Zeng et al. developed activated neutrophil membrane-coated nanoparticles, which disrupt PMN recruitment of neutrophils and simultaneously block neutrophil adhesion to CTCs as nano-baits, effectively reducing organ metastases in mice with breast cancer tumors (159). Micellar nanoparticles designed by Yang Long et al. (160) and Zhengze Lu et al. (161, 162), as well as the sponge-like nano-system studied by Chunyu Xia et al. (163), have shown efficacy in inhibiting pulmonary PMN formation by interfering with MDSC recruitment and mitigating MDSC-mediated immunosuppression. These innovations exemplify the potential for nanotechnology to reshape PMN-related immunotherapies. The eventual translation of these technologies into clinical applications is highly anticipated.

Additionally, emerging approaches such as leveraging gut microbiota to enhance immunotherapies (164), vaccine-based anti-tumor strategies (165), and TDEs-based immunotherapies (166) are under investigation, further broadening the horizon of PMN-targeted treatments.

### 4.3 Limitations

To the best of our knowledge, this study represents the first bibliometric assessment of current research on PMN. However, several limitations should be acknowledged. First, the literature data were exclusively sourced from the WOSCC database, potentially omitting relevant publications that were not indexed within this platform (such as in PubMed and Scopus database). Second, the analysis was limited to English-language literature, which may exclude significant studies published in other languages. Third, both original research articles and reviews were included in our analysis, which may have inflated the publication count for institutes that primarily produce reviews, thereby diluting the impact of original research. Lastly, publications from 2024 were not fully included, as the database was incomplete at the time of the data retrieval (September 18, 2024).

## 5 Conclusion

Over the past two decades, research in the field of PMN has witnessed a remarkable surge in activity. Significant contributions have been made by scholars from China and the United States, with Professor David Lyden of Cornell University recognized as the founder and pioneer of this field. Cornell University, Sichuan University, and Ruprecht Karls University Heidelberg stand out as the most prolific institutions driving advancements in PMN research. Key journals such as *Cancers* and *Cancer Research* have served as pivotal platforms for disseminating findings in this domain.

Furthermore, the prevailing research trends and advancements in the field were critically examined. Several key challenges persist in this discipline. First, the potential for PMNs to continue forming in distant organs after surgical resection of the primary tumor remains unclear. Accurate methods for detecting PMNs and determining which PMNs eventually lead to metastasis are still being explored. Moreover, the role of primary tumor heterogeneity in organ-specific PMN formation requires further investigation, as not all primary tumors may generate PMNs. The heterogeneity of EV cargo presents another challenge, necessitating strategies to precisely target tumor-associated EVs to inhibit PMN formation. Current PMN-related microbiome research primarily focuses on intestinal microorganisms, whereas the role of the tumor microbiome in PMN formation and immunotherapy remains underexplored. Although metabolic reprogramming is increasingly recognized as a key factor in cancer progression and metastasis, research on PMN immunometabolic reprogramming is still limited. A deeper understanding of its role in PMN formation is needed. Despite significant preclinical efforts to target PMNs, the effective translation of these findings into clinical applications remains uncertain. Continued research is necessary to bridge this gap. These challenges are still being addressed in basic and preclinical research, and have yet to be widely implemented in clinical practice. Emerging technologies are essential for advancing PMN research. Innovations in single-cell sequencing, spatial transcriptomics, liquid biopsies, advanced imaging techniques, nanotechnology, and artificial intelligence have significantly enhanced our understanding of the temporal evolution of organotropic metastasis. Integrating MPS-based *in vitro* models with large-scale omics analyses and *in vivo* genetic knockout models will be crucial for studying organotropic metastasis. Future research should focus on predicting metastatic risk and site-specific metastasis formation, preventing PMN-driven metastasis, and developing targeted therapeutic strategies.

Moving forward, topics like metastatic organotropism, extracellular vesicles, innate immunocytes (including macrophages and neutrophils), and immunotherapy are anticipated to remain at the forefront of PMN research, guiding the exploration of innovative solutions to combat cancer metastasis.

## Data Availability

The original contributions presented in the study are included in the article/**Supplementary Material**, further inquiries can be directed to the corresponding author/s.
