# Supplementary material for: Global hotspots and trends in pre-metastatic niche research: a bibliometric analysis(2005-2024)
Source: Front Immunol. 2025 May 29;16:1552053. doi: 10.3389/fimmu.2025.1552053 (PMC12159010; doi:10.3389/fimmu.2025.1552053)
Supplement: Supplementary file 1 [file Table1.docx]

Supplementary Tables

# Supplementary Tables

**Supplementary Table 1|** Top 10 journals in terms of number of frequency of co-citations, corresponding IF (2023) and JCR quartile.

| Rank | Cited-Journals | Co-citations | Country | IF (2023) | JCR Quartile |
| --- | --- | --- | --- | --- | --- |
| 1 | *Cancer Research* | 5,889 | USA | 12.5 | Q1 |
| 2 | *Nature* | 4,142 | United Kingdom | 50.5 | Q1 |
| 3 | *Cancer Cell* | 3,143 | USA | 48.8 | Q1 |
| 4 | *Nature Reviews Cancer* | 2,691 | United Kingdom | 72.5 | Q1 |
| 5 | *Cell* | 2,620 | USA | 45.5 | Q1 |
| 6 | *Proceedings of the National Academy of Sciences of the United States of America* | 2,476 | USA | 9.4 | Q1 |
| 7 | *Nature Communications* | 2,274 | United Kingdom | 14.7 | Q1 |
| 8 | *Nature Cell Biology* | 2,182 | United Kingdom | 17.3 | Q1 |
| 9 | *Plos One* | 1,986 | USA | 2.9 | Q1 |
| 10 | *Oncotarget* | 1,873 | USA | / | / |

**Supplementary Table 2**| Top 10 co-cited references involved in the field of PMN.

| Rank | Title | Journal | First author | Year | Co-citation | Type | DOI |
| --- | --- | --- | --- | --- | --- | --- | --- |
| 1 | Pre-metastatic niches: organ-specific homes for metastases | Nature reviews Cancer | Peinado H | 2017 | 223 | Review | 10.1038/nrc.2017.6 |
| 2 | Tumour exosome integrins determine organotropic metastasis | Nature | Hoshino A | 2015 | 161 | Article | 10.1038/nature15756 |
| 3 | Pancreatic cancer exosomes initiate pre-metastatic niche formation in the liver | Nature Cell Biology | Costa-Silva B | 2015 | 155 | Article | 10.1038/ncb3169 |
| 4 | Characteristics and Significance of the Pre-metastatic Niche | Cancer Cell | Liu Y | 2016 | 146 | Review | 10.1016/j.ccell.2016.09.011 |
| 5 | Cancer-derived exosomal miR-25-3p promotes pre-metastatic niche formation by inducing vascular permeability and angiogenesis | Nature Communication | Zeng ZC | 2018 | 94 | Article | 10.1038/s41467-018-07810-w |
| 6 | Tumor Exosomal RNAs Promote Lung Pre-metastatic Niche Formation by Activating Alveolar Epithelial TLR3 to Recruit Neutrophils | Cancer Cell | Liu YF | 2016 | 91 | Article | 10.1016/j.ccell.2016.06.021 |
| 7 | Minimal information for studies of extracellular vesicles 2018 (MISEV2018): a position statement of the International Society for Extracellular Vesicles and update of the MISEV2014 guidelines | Journal of Extracellular Vesicles | Théry C | 2018 | 86 | Review | 10.1080/20013078.2018.1535750 |
| 8 | Exosome-Mediated Metastasis: Communication from a Distance | Developmental Cell | Wortzel I | 2019 | 81 | Review | 10.1016/j.devcel.2019.04.011 |
| 9 | Effects of exosomes on pre-metastatic niche formation in tumors | Molecular Cancer | Guo YX | 2019 | 78 | Review | 10.1186/s12943-019-0995-1 |
| 10 | Shedding light on the cell biology of extracellular vesicles | Nature Reviews Molecular Cell Biology | van Niel G | 2018 | 78 | Review | 10.1038/nrm.2017.125 |
